# Pharmacogenetic Factors Shaping Treatment Outcomes in Chronic Obstructive Pulmonary Disease

**DOI:** 10.3390/genes16030314

**Published:** 2025-03-06

**Authors:** Charikleia Ntenti, Thomas Nikos Misirlis, Antonis Goulas

**Affiliations:** 1 First Laboratory of Pharmacology, School of Medicine, Aristotle University of Thessaloniki, 54124 Thessaloniki, Greece; 2 Special Unit for Biomedical Research and Education, School of Medicine, Clinical Research Unit, Aristotle University of Thessaloniki, 54124 Thessaloniki, Greece

**Keywords:** COPD, bronchodilators, inhaled corticosteroids, genetic variations, treatment response, pharmacogenetics, pharmacogenomics, personalized medicine

## Abstract

Chronic Obstructive Pulmonary Disease (COPD) manifests as a genetically diverse and intricate lung condition with various subtypes. The development of the disease and response to treatment are influenced by the interplay between genetic and environmental factors. The predominant therapeutic approaches include bronchodilator therapy and corticosteroid treatment. Studies in COPD pharmacogenetics involve genome-wide association (GWA) studies, gene profiling, whole-genome sequencing, and other omics-based investigations. Many of these investigations have focused on the association between genetic variations and the response to β2 agonist treatment. Additionally, several studies have explored the impact of gene variations on the response to inhaled corticosteroid (ICS) treatment, with a specific focus on polymorphisms in the glucocorticoid receptor (GR) signaling pathway. However, a significant challenge lies in the inconclusive or inconsistent results of these pharmacogenetic studies, underscoring the research community’s struggle to provide sufficient evidence for the clinical implementation of COPD pharmacogenetics. To address these challenges, further research and larger genome-wide studies are essential. These efforts aim to uncover additional COPD subtypes, identify predictors of treatment response, and discover novel genetic markers for COPD. The integration of genomics, detailed evaluations such as chest CT scans, spirometry tests, and blood analyses, along with DNA collection in clinical research, is critical for translating COPD pharmacogenetics into clinical practice. Furthermore, advancing our understanding of the complex interactions between genetics, phenotypes, and environmental factors will be pivotal for improving individualized prognostic assessments and enhancing treatment outcomes in COPD.

## 1. Introduction

Chronic Obstructive Pulmonary Disease (COPD) affects over 380 million people globally [1,2]. In 2021, it was the fourth leading cause of death worldwide, responsible for approximately 3.5 million deaths and accounting for about 5% of all global deaths. Notably, nearly 90% of COPD deaths in individuals under 70 occur in low- and middle-income countries. The prevalence of COPD is higher among men (15.70%) compared to women (9.93%), with the highest prevalence observed in the region of the Americas (14.53%) and the lowest in the South-East Asia/Western Pacific region (8.80%) [3].

COPD is a complex inflammatory lung condition characterized by irreversible airflow limitation, leading to breathing difficulties. The peripheral airways show an abnormal inflammatory response that can progress to respiratory failure [4]. COPD is marked by significant complexity and heterogeneity, presenting with diverse clinical features and varying degrees of severity. Initially asymptomatic, it typically evolves into persistent respiratory symptoms such as shortness of breath, cough, and sputum production [5]. While cigarette smoking is the primary risk factor, other environmental exposures also contribute to its development. As a progressive and dynamic disease, COPD exhibits variability in its morphological and clinical phenotypes, as well as in its severity and presentation [6]. This heterogeneity extends to airway inflammation, its responsiveness to anti-inflammatory therapies, systemic inflammation, and associated comorbidities.

The pathology in COPD patients commonly reveals features like chronic bronchitis, emphysema, bronchiectasis, or a combination thereof. Specifically, impaired gas transfer typically signifies lung parenchymal disease, generally associated with emphysema.

### 1.1. COPD Treatment

As previously mentioned, COPD is an intricate condition characterized by pathophysiological abnormalities, variability in disease severity, and diverse clinical characteristics. This heterogeneity leads to varied responses to medications among COPD patients. The primary objectives of COPD treatment are to mitigate disease progression, reduce the risk of exacerbations, and alleviate symptoms such as dyspnea, ultimately aiming to improve quality of life and decrease mortality [7,8]. For individuals with a low symptom burden, initial treatment typically involves short-acting bronchodilators (SABAs or SAMAs) used as needed to provide quick symptom relief [9]. However, as COPD progresses and symptoms become more persistent, regular long-acting bronchodilator therapy is recommended to maintain airway patency, improve lung function, and enhance daily activity levels. Long-acting β-2-agonists (LABAs) and long-acting muscarinic antagonists (LAMAs) serve as the foundation of maintenance therapy. These agents, which act via distinct mechanisms—LABAs enhance cAMP signaling, while LAMAs inhibit muscarinic receptor-mediated bronchoconstriction—are often combined to achieve greater bronchodilation [9,10,11]. Numerous randomized controlled trials (RCTs) have demonstrated that LAMA/LABA combinations offer superior therapeutic effects compared to monotherapies, providing enhanced symptom control and reducing the risk of exacerbation [12,13,14,15]. In cases where exacerbation prevention is a primary concern, inhaled corticosteroids (ICS) may be introduced, particularly in ICS/LABA combination therapy. This approach is especially beneficial for COPD patients with a history of frequent exacerbations and evidence of eosinophilic airway inflammation. In select patients, triple therapy (ICS/LAMA/LABA) appears to be more effective than dual bronchodilator therapy, offering improved symptom relief, lung function, and exacerbation reduction [16,17,18,19,20]. Although less commonly used today due to their narrow therapeutic window and side effects, methylxanthines (e.g., theophylline and aminophylline) remain an option for some COPD patients with persistent symptoms despite optimal bronchodilator therapy. These agents act as non-selective phosphodiesterase inhibitors, leading to bronchodilation and mild anti-inflammatory effects. However, their use has diminished due to a narrow therapeutic window and potential for significant adverse effects. A Cochrane review concluded that the available data do not support the use of methylxanthines for treating COPD exacerbations, citing modest and inconsistent benefits alongside a notable increase in adverse events like nausea and vomiting [9]. Some studies suggest that low-dose theophylline may have an additive benefit when used alongside ICS therapy by modulating airway inflammation. However, due to the risk of side effects such as arrhythmias, nausea, and drug interactions, methylxanthines are generally reserved for select patients who do not respond well to standard bronchodilator therapy. Recent guidelines suggest that while theophylline may offer some bronchodilatory benefits, its routine use is limited by the risk of toxicity and the necessity for regular monitoring. Consequently, methylxanthines are generally reserved for select patients who do not respond adequately to standard bronchodilator therapies [10].

Selective PDE4 inhibitors, such as roflumilast, have been developed to target the inflammatory processes central to COPD pathophysiology. By inhibiting the PDE4 enzyme, these agents reduce the breakdown of cyclic adenosine monophosphate (cAMP), leading to decreased inflammation. Clinical studies have demonstrated that roflumilast offers modest improvements in lung function and a reduction in exacerbation rates among COPD patients, particularly those with chronic bronchitis and a history of frequent exacerbations [11]. However, the therapeutic benefits of PDE4 inhibitors must be weighed against their side effect profile. Common adverse effects include gastrointestinal disturbances, such as nausea and diarrhea, as well as weight loss and headaches. These side effects can impact patient adherence and limit the widespread use of PDE4 inhibitors in COPD management [11].

Monoclonal antibodies (mAbs) have been extensively tested in pulmonary diseases, particularly asthma, with significant results. Anti-IgE and anti-IL-5 antibodies are now established as part of the standard treatment for severe asthma, with anti-IL-5 therapies proving highly effective in severe eosinophilic asthma. These findings led to the hypothesis that similar antibodies could also benefit a subset of COPD patients with high blood eosinophil counts. Consequently, mepolizumab and benralizumab, both targeting IL-5, have been tested in eosinophilic COPD patients [12].

In addition to pharmacological interventions, non-pharmacological therapies play a crucial role in moderate to severe COPD management. Pulmonary rehabilitation—a structured program involving exercise training, nutritional support, and patient education—has been shown to improve exercise capacity and quality of life [13]. For patients with chronic hypoxemia, long-term oxygen therapy (LTOT) is recommended, as it has been demonstrated to improve survival when used appropriately [14].

In very severe COPD, additional supportive measures become necessary. Non-invasive ventilation (NIV) can be beneficial in patients with chronic hypercapnic respiratory failure, as it enhances gas exchange and reduces hospitalizations. In specific cases, surgical interventions such as lung volume reduction surgery (LVRS) or even lung transplantation may be considered for patients who remain severely symptomatic despite optimal medical therapy [14].

### 1.2. Precision Medicine and Pharmacogenetics in COPD

Clinical practice guidelines categorize patients as members of groups with similar features and potentially common phenotypes, such as health status and disease progression [15]. However, this perspective fails to acknowledge the heterogeneity of the disease and individual characteristics. Recognizing that one size does not fit all, particularly in medical treatment, underscores the importance of early patient stratification in drug discovery. The selection and optimization of candidate drugs for well-defined patient subsets have the potential to inform the design of more rapid and targeted clinical trials [16,17].

The primary goal of precision medicine is to leverage appropriate biomarkers and genetic or other diagnostic tests to classify patients into specific subtypes. By identifying patients as responders or non-responders to a particular treatment, precision medicine can enhance the validity, efficacy, and utility of clinical practice. The evaluation of disease subtypes and genetic biomarkers in relation to proposed treatments enables drug developers to create more tailored treatments for these specific populations. Additionally, precision medicine aims for cost-effectiveness by reducing the necessary support from the healthcare system [18,19,20,21].

Pharmacogenomics and pharmacogenetics form integral components of precision medicine, where gene variations serve as biomarkers to assess whether an individual may exhibit a beneficial or harmful response to a specific treatment. The distinction between the terms “pharmacogenetics” and “pharmacogenomics” lies in the fact that pharmacogenetics primarily refers to the collective studies examining how a single gene polymorphism or gene variant correlates with medication response or side effects. Single nucleotide polymorphisms (SNPs), which are variations in a single nucleotide at a specific position, can impact the function or quantity of the protein (or mRNA) encoded by a gene, representing the most prevalent type of polymorphism in the human genome [22]. In contrast, pharmacogenomics explores a genome-wide spectrum to identify patients with diverse safety or efficacy outcomes before prescribing medication [23].

In the context of respiratory diseases, understanding the genomic foundation has led to more precise therapeutic approaches. Genomic medicine is considered a facet of precision medicine [24]. Given that gene regulation is often tissue-specific, comprehending the genetic control of lung-specific gene expression is crucial for understanding the heterogeneity of COPD. The GeneCards human gene database reports 522 genes involved in COPD pathogenesis [24]. For COPD pharmacogenomics studies, acknowledging disease heterogeneity is essential to integrate COPD into the framework of precision medicine. Unfortunately, only a limited number of pharmacogenetics (PGx) analyses have assessed treatment response in COPD, such as lung function or bronchodilator response, and no genetic variants have been identified that significantly predict treatment response [25].

## 2. Methodology

### 2.1. Search Strategy

A systematic search was conducted in the PubMed database with the aim of identifying all studies related to COPD treatment response and pharmacogenetic factors influencing the outcomes of the disease’s treatment. PubMed records spanning from 1968 to December 2024 were scrutinized using the search string (“pharmacogenetics” OR “pharmacogenomics” OR “genetic variation” OR “single nucleotide polymorphism” OR “SNP” OR “polymorphism” OR “genotype”) AND (“chronic obstructive pulmonary disease” [MeSH Terms] OR “COPD” OR “chronic obstructive lung disease”) AND (“treatment response” OR “drug response” OR “pharmacotherapy” OR “therapy” OR “medication”). The articles were manually reviewed, considering titles and abstracts, to identify relevant studies containing information on pharmacogenetic factors and COPD treatment responses and outcomes. Additionally, the bibliographies of the included articles and other pertinent articles within the databases were examined for further insights.

### 2.2. Inclusion Criteria and Data Collection

To be considered for inclusion, studies had to meet the following criteria: (a) falling into categories such as case–control, clinical trials, cross-sectional studies, or genome-wide association studies; (b) investigating the association between medication and genetic variants in relation to COPD treatment response; (c) furnishing information on genotype frequency within the study groups; and (d) offering adequate benchmarks for a COPD diagnosis. Additionally, only articles published in English were included.

### 2.3. Study Characteristics

**Figure 1:**
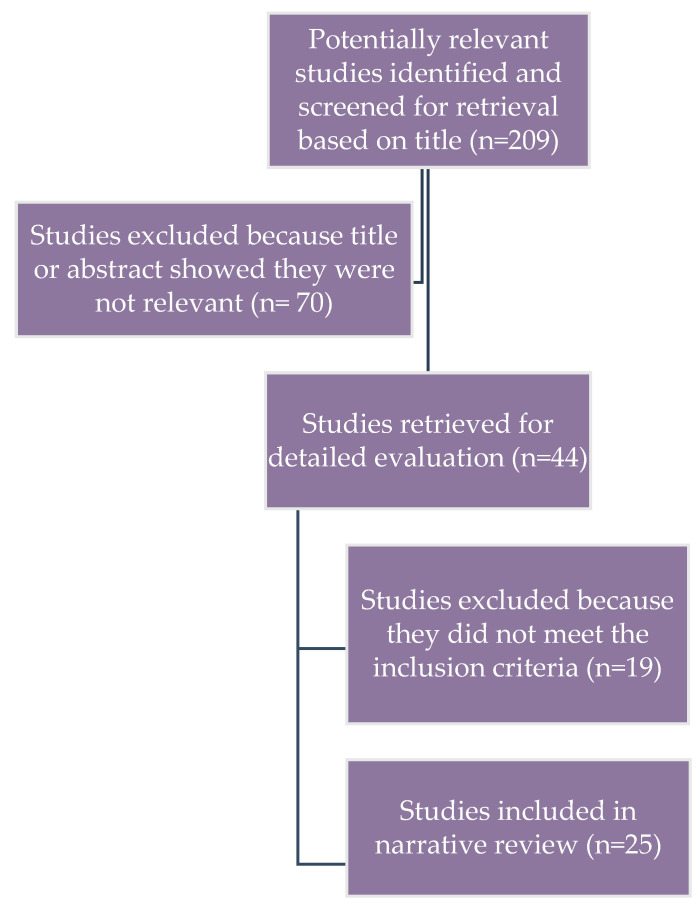
Flow diagram of study selection for the narrative review.

The database search resulted in 209 presumably relevant articles, of which 44, based on title and abstract relevance, were found to be appropriate for further evaluation. Of these, 19 studies were excluded because they did not meet the inclusion criteria. Thus, 25 studies of COPD treatment outcomes and pharmacogenetics were included in this narrative review (Figure 1). The database search was performed starting from 1968, but most of the studies were published after 2000, showing that a robust enhancement in genetic research has taken place in the last 20 years.

## 3. Results

### Pharmacogenetics Studies with Reference to Bronchodilating Response to β2 Agonists and Inhaled Corticosteroids

Out of the 25 studies investigating medication response in COPD and genetic variables, 19 studies delved into β2 agonist-related treatment responses in COPD. Among them, 10 exclusively focused on β2 agonist-related treatment responses while seven encompassed the use of β2 agonists in combination with ICS and/or anticholinergic drugs (Table 1). Regarding the examined genes, 15 studies specifically incorporated changes in the *ADRB2* genotype (Table 2).

Seven studies explored ICS-related treatment responses in COPD. Among them, three exclusively investigated ICS administration [39,40,42], while three assessed ICS in combination with a β2-agonist [20,41,49]. One study focused on LAMA treatment responses in COPD [43], and another investigated a therapeutic approach related to mepolizumab [38] (Table 3).

In the genetic association study conducted by Hizawa et al. [26], a cohort of 246 COPD patients, who were all participants in the “Hokkaido COPD cohort study” [51], underwent examination for short-term bronchodilator responses (BDRs) to salbutamol based on *ADRB2* genotypes Arg16Gly and Gln27Glu at codons 16 and 27, respectively. Spirometry was performed before and 30 min after salbutamol administration (bronchodilator therapy). The conclusive data indicated that the presence of the Arg16 allele was associated with lower bronchodilator responses to β2-agonist inhalation compared to patients homozygous for Gly16. No association was found between bronchodilator responses and the Gln27Glu genotype. The most frequent Arg16-Gln27 haplotype was significantly linked to decreased bronchodilator response to salbutamol.

In the study by Kim et al., 104 Korean patients with chronic obstruction, from the Korean Obstructive Lung Disease (KOLD) Cohort, were genotyped for codon 16 (Arg16Gly) and 27 (Gln27Glu) polymorphisms of the ADRB2 gene. Spirometry was performed, and immediate bronchodilator response was measured 15 min after inhalation of salbutamol. The study failed to demonstrate significant differences for codon 16 or codon 27 variants. Additionally, long-term response was assessed by observing spirometric changes before and 12 weeks after the inhalation treatment of a long-acting β2 agonist (salmeterol) combined with a glucocorticoid (fluticasone propionate) administered twice daily for 12 weeks. No association was found between effects on lung function (changes in bronchodilator response or FEV changes) and ADRB2 variants in Korean COPD patients [27].

Konno et al.’s study examined 189 COPD patients from the “Hokkaido COPD cohort study”. Bronchodilator response to two classes of bronchodilators, salbutamol or oxytropium bromide (anticholinergic), was measured every 6 months for 2 years. Arg16Gly and Glu27Gln genotypes were identified, and mean FEV1 values were evaluated as similar for salbutamol and oxytropium bromide. However, when participants were classified into two groups based on the bronchodilator causing the better response (salbutamol-dominant group and oxytropium-dominant group), the homozygous Gly16Gly genotype treated with salbutamol showed a tendency to have a higher FEV1 than when treated with oxytropium bromide. On the other hand, the presence of the Arg allele was significantly associated with an advantageous bronchodilator response to oxytropium. The study concluded that the preference of BDR for either β2-agonists or anticholinergics in COPD patients may rely on *ADRB2* polymorphism, and a possible combination of these two treatments might be more effective than monotherapy with only one drug category [29].

In a randomized, double-blind, double-dummy, parallel-group control trial conducted by Rabe and colleagues, the treatment effect of tiotropium or salmeterol on exacerbations was compared in a cohort of 5125 COPD patients genotyped for the SNPs rs1042713 (Arg16Gly) and rs1042714 (Gln27Glu). Patients with the Arg16Arg genotype treated with salmeterol had a significantly reduced exacerbation risk compared with the Arg16Gly and the Gly16Gly genotypes, suggesting a differential rs1042713 SNP (16 codon) effect on LABA administration response. Contrarily, no association was revealed between exacerbation risk and codon 16 variants in the tiotropium group. The study also identified a dependence on the use of ICS and the effect of Arg16Gly polymorphism on salmeterol treatment. Arg16Gly and Arg16Arg genotypes untreated with ICS at baseline showed remarkably prolonged time to the first exacerbation vs. the Gly16Gly genotype. No associations were found between the risk of exacerbation and variants at codon 27, regardless of the medication given [33].

Hussein et al. genotyped 115 Egyptian participants, including 61 COPD patients and 54 controls, for two SNPs, rs1042713 (Arg16Gly) and rs1042714 (Gln27Glu). The bronchodilator response to a β2-agonist (salbutamol) was associated solely with the Gln27Glu genotype, with the Glu27Glu variant carrying patients showing the maximum response. The study concluded that ADRB2 gene polymorphisms may play a significant role in bronchodilator response, but only narrowly among the Egyptian population [37].

In two large, randomized phase III studies, each lasting 26 weeks, 648 COPD patients were genotyped for four polymorphisms in the ADRB2 gene: Arg16Gly, Thr164Ile, Gln27Glu, and a variant in the 5′ untranslated region (rs1042711). Both trials, as reported by Yelensky et al., evaluated FEV1 after 12 weeks of indacaterol administration as the primary endpoint, along with additional endpoints. While indacaterol demonstrated superiority over placebo in LABA treatment, there was little evidence of a significant association between ADRB2 variants and bronchodilator response (FEV1, transitional dyspnea index, exacerbations), suggesting that genetic variation in ADRB2 does not appear to influence the BDR response to LABA treatment in COPD patients [31].

Bleecker et al. conducted a study involving two datasets from separate double-blind trials: a 12-month trial (NCT00206167) with 1483 patients, and a 6-month trial (NCT00206154) with 1383 patients, totaling 2866 COPD patients genotyped for the Arg16Gly variant. Participants with moderate to very severe COPD were randomized to receive either budesonide (glucocorticoid) alone, budesonide combined with formoterol (LABA), or a placebo. The study examined the impact of the Arg16Gly genotype on pre-dose and post-dose FEV1, exacerbations, and adverse effects. No significant association was found between the Arg16Gly genotype and therapeutic response or tolerability to ICS alone or combined with a LABA [30].

In a study by Mokry et al., 107 Slovakian patients with acute exacerbation COPD (AECOPD) were genotyped for Arg16Gly and Gln27Glu polymorphisms in the ADRB2 gene using allele-specific PCR. Patients were categorized into two groups: Gly16/Glu27-positive (76 individuals) and Gly16/Glu27-negative (31 individuals). Bronchodilator response and lung function after salbutamol administration were evaluated via body plethysmography. Significant improvements in FEV1, FVC, and peak expiratory flow (PEF) were observed in both groups. The authors concluded that different ADRB2 haplotypes may influence lung function and disease progression, although no immediate bronchodilator response to salbutamol was observed in AECOPD patients [28].

Sayers et al. investigated the pharmacological effects of indacaterol in recombinant cells expressing common SNPs and haplotypes of the β2-adrenoceptor, as well as in human airway smooth muscle (ASM) cells. Indacaterol demonstrated high efficacy in cells transfected with human β2-adrenoceptor variants, regardless of expression level, but showed lower efficacy in ASM cells. Among LABAs, indacaterol’s response fell between salmeterol and formoterol in terms of efficacy [48].

In a crossover study, Mochizuki et al. examined the effects of two LABAs—salmeterol (inhaled) and tulobuterol (patch)—on 36 COPD patients over 12 weeks. The study assessed changes in lung function (FEV1, %FEF25–75%, IC/TLC) and 6-min walk distance (6MWD) in groups divided by the CysGlyGln genotype. Patients with zero or one copy of the haplotype showed greater improvement compared to those homozygous for CysGlyGln, suggesting that the homozygous CysGlyGln haplotype may be associated with LABA desensitization in COPD [45].

Condreay et al. conducted a pharmacogenomics study examining 17 clinical trials involving 6705 COPD patients treated with umeclidinium (UMEC), vilanterol (VI), combination therapy, or placebo. They genotyped patients for *ADRB2* polymorphisms, HLA alleles, and 298 SNPs. No significant associations were found between genetic variants and therapeutic response, suggesting genetic factors do not strongly influence responses to LABA, LAMA, or combination therapy in COPD [34]. Additionally, Condreay et al. conducted a genome-wide association study (GWAS) analyzing data from 10 clinical studies involving 2005 subjects with COPD. The study found that common genetic variants did not significantly influence the response to fluticasone propionate/salmeterol treatment. However, a low-frequency variant on chromosome 20 was associated with improvements in the St. George’s Respiratory Questionnaire (SGRQ) scores, indicating a potential link to quality of life enhancements [47].

Kim et al. investigated six candidate genes in 389 severe COPD patients from the National Emphysema Treatment Trial (NETT). They identified associations between bronchodilator responsiveness and SNPs in *EPHX1*, *SERPINE2*, and *ADRB2*, with rs1009668 in *EPHX1* being significantly linked to reduced bronchodilator response [46]. In a GWAS meta-analysis by Hardin et al. involving 5789 COPD patients, associations between bronchodilator response and SNPs in *KCNJ2*, *CDH13*, and *GOLGA8B* were identified, though the results lacked genome-wide significance. These findings highlight the complex genetic landscape influencing bronchodilator response in COPD [35].

Fawzy et al. identified a significant association between the *MIR-196a2* rs11614913 polymorphism and salbutamol responsiveness in Egyptian COPD patients. Patients with the TT genotype exhibited the highest bronchodilator response, while CC genotype carriers showed the lowest, suggesting the potential of *MIR-196a2* as a pharmacogenetic marker [36].

Panebra et al. identified eight common haplotypes derived from 26 polymorphisms across the ADRB2 gene, including the promoter, 5′UTR, coding, and 3′UTR regions. Whole-gene transfection experiments using COS-7 cells revealed four haplotypes associated with ADRB2 protein expression and agonist-promoted downregulation on the cell surface. The expression and downregulation of ADRB2 protein were found to be haplotype-specific. Two haplotypes exhibited higher protein expression and reduced downregulation, indicating better clinical outcomes. These findings suggest that the functional effects of *ADRB2* allelic variations arise from combinations of polymorphisms rather than individual SNPs, providing a more refined approach for identifying clinical subtypes in pharmacogenomic studies [52].

Obeidat et al., conducted a pharmacogenomic GWAS using data from the Lung Health Study-2 (LHS-2), an RCT assessing inhaled corticosteroids (ICS) on FEV1 decline over three years. The study included 1,116 COPD patients randomized to triamcinolone (559) or placebo (557), with spirometry every six months. For GWAS, 802 LHS-2 participants were genotyped to assess genotype-ICS response association. Validation was performed using 199 COPD patients from the Advair, Biomarkers in COPD (ABC) trial, randomized to ICS (fluticasone) or placebo. Five loci were linked to ICS response, with rs111720447 on chromosome 7 replicated. Results showed COPD patients carrying the A allele in rs111720447 who received ICS had greater FEV1 decline than those on placebo. ENCODE data revealed rs111720447 is near glucocorticoid receptor (GR) binding sites in dexamethasone-treated A549 alveolar cells. While the variant does not alter gene expression, its location suggests a structural or charge effect on the GR complex [42].

Although all previously studied variations have focused on pharmacodynamic genes, polymorphisms in pharmacokinetic genes can also play a crucial role in individual responses to medications. However, these associations are extremely difficult to identify due to the complexity of pharmacokinetic factors that influence drug levels [53]. Variations in the *CYP1A2* gene, for example, can alter enzyme activity, directly impacting the metabolism of theophylline. A study examining *CYP1A2* gene polymorphisms found that patients with asthma and COPD who carried specific alleles exhibited significantly reduced theophylline clearance [32]. However, another relevant study conducted solely in asthmatic patients reported opposite findings [54].

## 4. Discussion

To evaluate the role of pharmacogenetics in COPD therapy, we conducted a comprehensive review of 25 studies involving 37,022 individuals. Our analysis focused on the association between the most commonly used COPD medications—β2-agonists and inhaled corticosteroids (ICS)—and genetic variants that influence treatment response and disease susceptibility.

A total of 15 studies examined whether functional variants in the β2-adrenergic receptor (*ADRB2*) gene are linked to bronchodilator response and lung function improvement in COPD patients. The three most studied loci were Arg16Gly (rs1042713), Gln27Glu (rs1042714), and Thr164Ile (rs1800888). Additionally, six other studies explored the influence of polymorphisms in genes such as *HLA, EPHX1, SERPINE2, MAPK8, AZU1, KCNJ2*, *CDH13*, and *MIR196-a2* on β2-agonist treatment response. Despite extensive research, inconsistent findings emerged regarding the impact of specific SNPs or haplotypes on bronchodilation. While one study reported a positive association between the Arg16Arg genotype and treatment response, two others suggested a negative correlation. Additionally, six trials found no significant association between *ADRB2* genetic variants and bronchodilator response, three of which were well-powered studies. The conflicting findings in β2-agonist-related studies could be attributed to multiple factors. Small sample sizes have often led to underpowered studies, limiting the ability to detect true associations. Differences in trial designs may have contributed to variability in findings, while the presence of environmental factors, such as smoking status and disease comorbidities, may have influenced treatment responses. Furthermore, there were multiple polymorphisms in *ADRB2* results in varied haplotype distributions based on individual characteristics such as sex, ethnicity, and smoking history, which could explain the variation in bronchodilator response. Apart from bronchodilation, *ADRB2* genetic variations may also alter receptor desensitization and agonist-promoted downregulation, adding further complexity to the pharmacogenetic landscape of β2-agonists in COPD treatment.

However, the reviewed studies provided inconsistent results about the role of particular SNPs or haplotypes in the bronchodilating action of β2 agonists. Some studies had small sample sizes, increasing the risk of unreliable conclusions. Additionally, six trials found no association between identified genetic variants and medication therapy response, while three were well-powered. Conflicting results in β2 agonist-related studies may stem from underpowered studies, differences in trial designs, and the presence of concurrent variables such as environmental factors (smoking status) or disease comorbidities.

Furthermore, parallel polymorphisms on specific genes generate various *ADRB2* haplotypes, and potential interactions between different variants around and inside the *ADRB2* gene could yield inconsistent outcomes. The studies indicated that *ADRB2* haplotypes occur at different frequencies based on various patient characteristics, such as sex, ethnicity, and smoking status. Genetic variations in the *ADRB2* gene, apart from influencing bronchodilation, may also impact desensitization and agonist-promoted receptor downregulation. Advancing our understanding of how changes in gene expression or function affect clinical phenotypes will be key to translating *ADRB2* genetic insights into practical applications in clinical practice.

Eight of the studies included in this review examined the influence of genetic variation on ICS response. Six studies focused solely on ICS-related polymorphisms, while two explored the impact of genetic variation on treatment with combined therapies, including ICS and β2-agonists or ICS, β2-agonists, and LAMAs. The *GLCCI1* gene was assessed in three studies, with one study identifying a strong correlation between *GLCCI1* polymorphisms and ICS response, while the other two reported minor or no association. Additional SNPs in genes such as *NR3C1*, *GSTM3*, *PSMD8*, *FKBP5*, *ALOX5AP*, *CYP2E1*, and *SMAD3* were also linked to ICS responsiveness. Among these, *FKBP5* and *PSMD8* polymorphisms demonstrated measurable effects on ICS efficacy, while a large-scale study identified a novel variant (rs111720447) near the glucocorticoid receptor gene, which was significantly correlated with ICS response in COPD patients.

It is well established that pharmacogenetic research in COPD has largely focused on *ADRB2* polymorphisms and how they affect bronchodilator response [55]. On the other hand, studies exploring genetic factors influencing the response to LAMAs—the most common bronchodilators—are still quite limited. One noteworthy study is by Umeda et al., which looked at the *ADRB2* Arg16Gly polymorphism and its impact on the effectiveness of tiotropium. Their findings suggested that patients with the Arg16 homozygous genotype showed a significant improvement in FEV1 over the course of treatment. However, this study did not directly examine genetic variations in muscarinic receptors—it focused again on *ADRB2* instead. At this point, there is no strong evidence linking muscarinic receptor gene polymorphisms to differences in how patients respond to LAMA therapy in COPD [43].

Similarly, there is limited direct evidence linking specific polymorphisms mAb efficacy in COPD. However, studies in asthma and other inflammatory diseases suggest that genetic variations in cytokines such as IL-5, IL-4, IL-13, and TNF may influence responses to biologic therapies [56,57,58].

## 5. Conclusion & Future Directions

COPD is a heterogeneous disease where precision medicine holds promise through genetics, omics research, and biomarkers [59]. While significant morbidity and mortality persist, pharmacogenetics research has advanced over the past 15 years. However, the current therapeutic options are just starting to incorporate subtype-directed treatment decisions for patients [60]. The success of subtype-dependent therapies has been modest, partly due to the unclear categorization of different phenotypes (such as emphysema or airway disease) and the limited range of COPD medication classes, which generally yield moderate effects [61].

In pharmacogenetics and genetic research, the primary goal is to link genetic variants with specific COPD phenotypes, providing insights into distinct disease subtypes [44]. Furthermore, the pharmacogenetic perspective seeks to correlate these specific subtypes with precise treatments, enhancing medication outcomes while minimizing side effects [62]. Current literature highlights the growth of discovery-driven research, such as genome-wide association studies, gene expression profiling, whole-genome sequencing, and other omics approaches. These efforts focus on integrating pharmacogenetic traits with diverse omics data to address the challenges posed by limited sample sizes [63]. One of the key challenges in COPD pharmacogenetics is the disease’s complexity, compounded by small sample sizes, limited access to relevant tissues, and inconsistencies in clinical trial outcomes across different medications. Additionally, most studies have focused on European populations, overlooking genetic variability in non-European groups. However, it is essential to recognize the existence of distinct bronchodilator response phenotypes among Caucasians, African Americans, and non-Hispanic white individuals. Given that COPD is a global health concern, future research endeavors should encompass non-European populations to ensure a comprehensive understanding. Moreover, the consideration of publication bias is crucial, as studies without identified associations may often go unpublished.

At the same time, genome-wide association studies (GWAS), gene expression profiling, and other omics approaches have become central to COPD research. These methodologies aim not only to link genetic variants with specific disease subtypes but also to optimize treatment strategies. Yet, despite these advancements, integrating pharmacogenetic insights into clinical practice remains challenging. To overcome this hurdle, larger sample sizes, robust phenotype assessments (e.g., spirometry, CT scans, blood biomarkers), and biobank data must be incorporated. In this regard, collaborative efforts between industry and academia will be critical to bridge this gap and enable genotype-guided clinical trials. Furthermore, as drug development for COPD continues to evolve, it is important to recognize that new formulations and delivery methods (e.g., inhalers, nebulizers, oral, injection) are constantly emerging. Therefore, studying the genetic basis of therapeutic responses in early clinical trials could help identify pharmacogenetic effects and justify genotype-guided approaches. Despite this potential, current studies have yet to yield clinically actionable results. Ultimately, moving forward, deeper exploration of genotype–phenotype interactions—considering both genetic and environmental factors—will be key in optimizing individualized treatment. In doing so, researchers can improve outcomes and elevate healthcare standards for COPD patients. Currently, our comprehension of COPD genetics is incomplete, and the practical integration of pharmacogenetics into COPD clinical practices remains uncertain. Further investigation, along with expanded genome-wide studies involving larger cohorts, is imperative to unveil additional COPD subtypes, predictors of drug responses, and novel genetic biomarkers associated with COPD. Advancing pharmacogenetics research demands a more thorough exploration of the diverse nature of COPD pathology to ensure the precise application of suitable treatments for individual patients. Moreover, identifying responders to newly developed or modified drug formulations will aid in discerning phenotype-specific COPD pathophysiology. It may not be entirely feasible to rely solely on genetics for accurately gauging disease susceptibility and treatment responses in patients. Progressing further involves deepening our understanding of the simultaneous interactions between genotype and phenotype, encompassing both genetic factors and environmental influences. For instance, the effectiveness of COPD therapy may be influenced by sex, but the current evidence remains heterogeneous. Studies suggest that ICS and ICS/LABA combinations provide similar lung function benefits in both men and women. However, when it comes to exacerbation prevention, PDE4 inhibitors appear to be more effective in men than in women, while azithromycin shows equal effectiveness in both sexes. Additionally, no significant sex-related differences have been observed in the efficacy of muscarinic antagonists for disease control. Despite these findings, an important limitation in COPD pharmacogenetics is the gender imbalance in clinical trials, where enrollment ratios between men and women are often skewed. This discrepancy may introduce sex bias in measured treatment outcomes, making it difficult to draw definitive conclusions about the role of sex in COPD therapy response [64].

COPD represents a diverse condition where precision medicine can be applied, leveraging genetics, omics research, and diverse biomarkers [59]. The success of subtype-dependent therapies has been modest, partly due to the unclear categorization of different phenotypes (such as emphysema or airway disease) and the limited range of COPD medication classes, which generally yield moderate efficiency. Genome-wide association studies have run concurrently with clinical trials evaluating novel COPD treatment drugs and existing therapies, covering a spectrum from LABA, LAMA, and ICS to oxygen therapy. These studies assess medication responses concerning COPD, including lung function decline, comorbidities such as COPD-asthma overlap, and other relevant outcomes.

Gene-expression profiling stands out as a crucial complementary process, distinguishing molecular subtypes within the COPD spectrum [65]. To effectively translate COPD pharmacogenetics into clinical practice and facilitate genotype-guided clinical trials for COPD therapies, studies must incorporate genomic samples, comprehensive phenotype assessments (such as chest CT scans, spirometry tests, blood counts), and DNA collection [66]. Biobank studies are poised to provide larger sample sizes, offering enhanced insights into COPD genetics, spanning treatment responses and susceptibility, including genomic biomarkers linked to specific clinical phenotypes [67,68]. Consequently, the integration of genetic findings from pharmacogenetics studies into electronic medical records becomes imperative, furnishing essential information on therapeutic outcomes and medication administration. Moreover, large-scale COPD pharmacogenetics studies necessitate collaborative efforts between the industry sector and academic partners.

## Figures and Tables

**Table 1:** Baseline characteristics and primary endpoints of COPD studies.

Author	Country	Number of Participants	Age	Ethnicity/Race	Primary Endpoint
Hizawa et al., 2007 [26]	Japan	246	>40 years	Japanese	BDR, FEV1, FEV1% predicted changes
Kim et al., 2008 [27]	South Korea	104	65 years mean	South Korean	BDR
Kim et al., 2008 [27]	South Korea	389	67 years mean	South Korean	FEV1, FEV1% predicted changes
Mokry et al., 2008 [28]	Slovakia	107	66 years mean	Slovakian	FEV1, FVC changes, PEF values
Konno et al., 2011 [29]	Japan	184	>40 years	Japanese	BDR
Bleecker et al., 2012 [30]	USA	2866	65 years mean	Mixed	FEV1% predicted changes, exacerbations
Yelensky et al., 2012 [31]	USA	648	>40 years	Mixed	FEV1, PEF, exacerbations
Mochizuki et al., 2012 [32]	Participants from 10 countries	36	>40 years	White, Asian	FEV1% predicted, FVC changes, 6MWD
Rabe et al., 2014 [33]	Participants from 25 countries	5125	63 years mean	Caucasians	exacerbations
Condreay et al., 2016 [34]	Ν/A	6075	63 years mean	Mixed	FEV1, FVC, FEV1/FVC changes
Hardin et al., 2016 [35]	Data from four clinical trials	5789	63 years mean	Caucasian, African American	BDR, FEV1 changes
Fawzy et al., 2016 [36]	Egypt	224	58 years mean	Egyptian	BDR, FEV1 changes
Hussein et al., 2016 [37]	Egypt	115	61 years mean	Egyptian	BDR, FEV1 changes
Condreay et al., 2019 [38]	Data from 10 clinical trials	2005	>40 years	Mixed	FEV1 changes, SGRQ score changes
Hosking et al., 2021 [25]	Data from 23 clinical studies and two disease cohorts	8439	>40 years	all ethnicities (83% White European)	Exacerbation rate, FEV1 changes, SGRQ score
Lee SW et al., 2018 [39]	Taiwan	74	69 years mean	Taiwanese	FEV1, FVC changes, symptoms
Yuan Lei et al., 2017	China	204	67 years mean	Chinese	BDR, FEV1 changes
Mosteller et al., 2017 [40]	Data from three studies	465	61 years mean	Caucasian	FEV1, FEV1% predicted changes
Russo et al., 2019 [41]	Italy	71	73 years mean	Italian	FEV1% predicted changes, SGRQ score, 6MWD
Obeidat et al., 2019 [42]	Sample from multicenter study (LHS-2)	802	55 years mean	Caucasian	FEV1 changes
Umeda et al., 2008 [43]	Japan	44 (COPD)	>40 years	Japanese	FEV1 and FEV1% changes, SGRQ score changes

**Table 2:** COPD studies showing association between β2 agonist medication response and common genetic variants.

Study	Treatment	Result
Studies showing association		
Hizawa et al. [44]	SABA	Arg16 allele was associated with lower bronchodilator response to SABA. No association between BDR and codon 27 variant
Konno et al. [29]	SABA (or LAMA)	Arg16 allele was associated with lower bronchodilator response to SABA
Rabe et al. [33]	LABA	Arg16 allele was associated with reduced exacerbation risk
Hussein et al. [37]	SABA	Glu27Glu variant was associated with higher bronchodilator response to SABA. No association between BDR and codon 16 variant
Mochizuki et al. [45]	LABA	CysGlyGln haplotype carriers connected with LABA desensitization
Kim et al. [46]	SABA	EPHX1 variants associated with bronchodilator response
Hardin et al. [35]	SABA	No association between ADRB2 variants and BDR. SGCD and GOLGA8B gene variants associated with response to β2 agonists
Fawzy at al. [36]	SABA	MIR-196a2 gene variant associated with response to β2 agonists
Studies showing no association		
Kim et al. [27]	SABA	No association between ADRB2 variants and acute BDR or 12-week change in FEV1
Yelensky et al. [31]	LABA	No association between codon ADRB2 variants and FEV1 change, dyspnea index or exacerbations
Bleecker at al. [30]	LABA (+ICS)	No association between ADRB2 variants and FEV1 change or exacerbations
Mokry et al. [28]	SABA	Haplotypes of codon 16 and 27 variants were not associated with bronchodilator response
Condreay et al. [34]	LABA (+LAMA)	No association between ADRB2 variants, HLA alleles or other SNPs and bronchodilator response
Condreay et al. [47]	LABA (+ICS)	Common genetic variants are not associated with FEV1 change, exacerbation rate or QoL status
Sayers et al. [48]	LABA	No significant genotype-dependent effects were found for common variants examined.
Hosking et al. [25]	LABA (+ICS)	No association between common genetic variants and AECOPD treatment response

ADRB2: Adrenergic Receptor β2; AECOPD: Acute Exacerbation Chronic Obstructive Pulmonary Disease; BDR: Bronchodilator responsiveness; FEV1: Forced expiratory volume in 1 s; ICS: Inhaled corticosteroid; LABA: Long-acting b-agonist; LAMA: Long-acting muscarinic antagonist; QoL: Quality of Life; SABA: Short-acting b-agonist; SNP: Single-nucleotide polymorphism.

**Table 3:** COPD studies showing association between ICS medication response and common genetic variants.

Study	Treatment	Result
Studies showing association		
Lee et al. [49]	ICS (+LABA)	GLCCI1 GG genotype was associated with impaired corticosteroid efficacy
Lee et al. [39]	ICS	*PSMD8* gene polymorphism associated with differential response to ICS
Russo et al. [41]	ICS (+SABA or LAMA)	*FKBP5* gene GA genotype was associated with higher lung function improvement and better SGRQ score—*GLCCI1* (rs37972) TT carriers showed higher lung function improvement (small sample)
Obeidat et al. [42]	ICS	An allele in rs1117520447 SNP was associated with higher FEV1 decline rate
Cowan et al. [20]	ICS (+roflumilast or LABA)	*SMAD* gene GG genotype was associated with better ICS treatment response–CYP2E1 gene with at least one copy of reference allele was associated with better ICS treatment response
Kim et al. [50]	ICS (+LABA)	GG carriers of *CRHR1* responded better to ICS (+LABA) compared to heterozygotes
Studies showing no association		
Mosteller et al. [40]	ICS	No association between *GLCCI1* and response to ICS

FEV1: Forced expiratory volume in 1 s; ICS: Inhaled corticosteroid; LABA: Long-acting b-agonist; LAMA: Long-acting muscarinic antagonist; SABA: Short-acting b-agonist; SGRQ: St George respiratory questionnaire.

## Data Availability

No new data were created or analyzed in this study. Data sharing is not applicable to this article.
